# Quantitative Evaluation of Thermal Ageing State of Cross-Linked Polyethylene Insulation Based on Polarization and Depolarization Current

**DOI:** 10.3390/polym15051272

**Published:** 2023-03-02

**Authors:** Ping Huang, Wenyao Yu, Chunhao Lu, Xinghua He, Yiyi Zhang, Yansong Liu, Jiaheng Zhou, Yuwang Liang

**Affiliations:** 1 Guangxi Power Transmission and Distribution Network Lightning Protection Engineering Technology Research Center, Guangxi University, Nanning 530004, China; 2 Electric Power Research Institute, Guangxi Power Grid Corporation, Nanning 530002, China; 3 SPIC Guangxi Electric Power Co., Ltd., Nanning 530003, China

**Keywords:** cross-linked polyethylene (XLPE), polarization and depolarization current (PDC), elongation at break retention rate (ER%), stable relaxation charge quantity, dissipation factor at 0.1 Hz

## Abstract

The widespread use of cross-linked polyethylene (XLPE) as insulation in cables may be attributed to its outstanding mechanical and dielectric properties. In order to quantitatively evaluate the insulation status of XLPE after thermal ageing, an accelerated thermal ageing experimental platform is established. Polarization and depolarization current (PDC) as well as elongation at break of XLPE insulation under different ageing durations are measured. XLPE insulation state is determined by the elongation at break retention rate (ER%). Based on the extended Debye model, the paper proposed the stable relaxation charge quantity and the dissipation factor at 0.1 Hz to evaluate the insulation state of XLPE. The results show that the ER% of XLPE insulation decreases with the growth of ageing degree. The polarization and depolarization current of XLPE insulation will increase obviously with thermal ageing. Conductivity and trap level density will also increase. The number of branches of the extended Debye model increases, and new polarization types appear. Stable relaxation charge quantity and dissipation factor at 0.1 Hz proposed in this paper have a good fitting relationship with ER% of XLPE insulation, which can evaluate the thermal ageing state of XLPE insulation effectively.

## 1. Introduction

A power cable is one of the most important equipment for the transmission and distribution of electric energy in power systems. Its normal operation is the key factor for the safety and stability of the power grid. Cross-linked polyethylene (XLPE) cables are widely used in urban power networks due to their great electrical, mechanical, and physical properties [1]. The XLPE insulation layer plays a decisive role in the safe and reliable operation of the power cable. As most cables are laid underground, the laying mode and environment are harsh. Under the influence of heat, water, electricity, and mechanical stresses, the XLPE insulation layer of cables will gradually deteriorate, which will lead to changes in the dielectric properties of the XLPE [2,3]. Among various stress influences, thermal stress is one of the most important factors affecting the operation and insulation performance of XLPE cable [4]. The allowable maximum working temperature of XLPE cable is 90 °C. When an overload or a short circuit happens, the insulation temperature of the cable can rise to 250 °C [5]. Therefore, during the operation of the cable, it is inevitable to suffer long-term thermal stress, under which the XLPE insulation will suffer irreversible damage. The thermal stress will also affect the formation and growth of water tree and electric tree indirectly [6,7].

Thermal ageing results in some physicochemical changes of XLPE. However, using these methods to evaluate the cable insulation status requires expensive equipment and the experiment is often destructive, which is not suitable for the field application. The detection of cable insulation should be non-destructive. Nowadays, the major non-destructive detection methods include partial discharge, dissipation factor, insulation resistance, etc [8,9,10]. These methods are widely used to test the insulation status of the XLPE cable in the field, but there are some shortcomings in these methods. For instance, the signal of partial discharge in the insulation of the on-site cable is weak, the electromagnetic interference is strong, and is affected by the sensitivity of the sensor and signal attenuation, so it is difficult to extract and identify the partial discharge signal. Dissipation factor values at low frequency are usually used to assess the insulation status of XLPE. However, the dissipation factor measurement at low frequency takes a longer time. The insulation resistance measurement result needs to be temperature corrected. In recent years, the dielectric response method, which takes dielectric as the test object, as a novel non-destructive insulation evaluation technology, has become a research hotspot in laboratories and the field. It mainly includes a time-domain dielectric response method and frequency-domain dielectric response method. The time-domain detection methods mainly include IRC [11,12], PDC [3,13], and RVM [14]. The main detection method in the frequency domain is FDS [15,16]. The polarization and depolarization current (PDC) method has been applied in the field of insulation because of its characteristics such as simple equipment, convenient operation, and a large amount of information reflected. Leibfried et al. believed that the PDC method can test insulation materials in the time domain, and as long as the time is long enough, PDC method will reflect rich dielectric information [17]. Mishra et al. extracted the de-trapping current from PDC and found that the time constant of the de-trapping current is related to paper conductivity, oil conductivity, dissipation factor, and insulation life [18]. Ye et al. analysed the relationship between cable water tree ageing and PDC, and tanδ obtained from time domain conversion to frequency domain can also effectively describe the degree of cable water tree ageing [3]. Mursalin et al. analysed the extended Debye model of three-branch and four-branch to calculate the loss factor for numerical simulation, and the results show that the four-branch R-C branch has better results [19]. However, few scholars focus on the dielectric properties of XLPE insulation after thermal ageing, and there is a lack of quantitative evaluation of the state of XLPE insulation after thermal ageing.

In this paper, the XLPE disk and dumbbell samples are prepared, and then the XLPE samples are subjected to thermal ageing with a temperature of 150 °C and a duration of 48 days in the air. The mechanical property and PDC of XLPE insulation under different thermal ageing times are measured. The effects of thermal ageing on the polarization and depolarization current value, conductivity, trap energy level, and elongation at break retention rate (ER%) of XLPE insulation samples are analysed. Finally, some characteristic parameters of XLPE insulation under different thermal ageing durations are studied by using the extended Debye model. The stable relaxation charge quantity and the dissipation factor at 0.1 Hz are proposed to evaluate the thermal ageing state of XLPE insulation.

## 2. Basic Theory

### 2.1. Principle of PDC Method

In recent years, PDC has been widely used as a time-domain detection method in the diagnosis of transformer oil-paper insulation and XLPE cable insulation. According to the principle of dielectric response, when the electric field generated by the external voltage source is applied to the uniform dielectric, the total current through the dielectric is superimposed by the displacement current and the conductance current, which can be expressed as:

(1) $$i(t)=C_{0}[\frac{\sigma }{\varepsilon_{0}}u(t)+\varepsilon_{r}\frac{du(t)}{dt}+\frac{d}{dt}\int_{0}^{t}f(t-\tau )u(\tau )d\tau ]$$

where *C*_0_ is the geometric capacitance of the sample, *σ* is the conductivity of the sample, *ε_0_* and *ε_r_* are the vacuum dielectric constant and the relative dielectric constant of the measured sample, respectively, and ƒ(*t*) is the dielectric response function of the measured sample.

**Figure 1:**
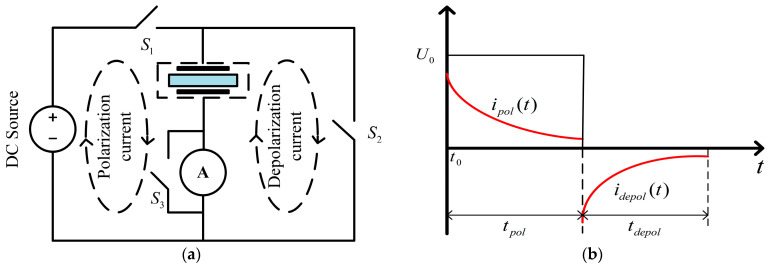
(**a**) Measuring circuit diagram; (**b**) Principle for the PDC measurement.

As shown in Figure 1a, when switch *S*_1_ is closed, DC excitation applies an electric field to the dielectric under test. Under a constant electric field, the measured dielectric begins to charge with different time constants. Assuming that it is fully charged, the current generated by this process is the polarization current, which can be expressed as:

(2) $$i_{pol}(t)=C_{0}U_{0}\left[\frac{\sigma_{0}}{\varepsilon_{0}}+f(t)\right]$$

After charging the dielectric for a while, *S*_1_ is opened, *S*_2_ is closed, and the DC excitation is removed. The current generated in this process is the depolarization current, which can be expressed as:

(3) $$i_{dep}(t)=-C_{0}U_{0}\left[f(t)-f(t+t_{pol})\right]$$

where *U*_0_ is the applied DC excitation voltage, and *t_pol_* is the polarization time.

The switch *S*_3_ protects the measuring device. Polarization current and depolarization current are always measured by an electrometer or a high-sensitivity ammeter. In this paper, DIRANA is used to measure currents. The schematic diagram of polarization and depolarization current generated by the PDC process is shown in Figure 1b.

### 2.2. Extended Debye Model

**Figure 2:**
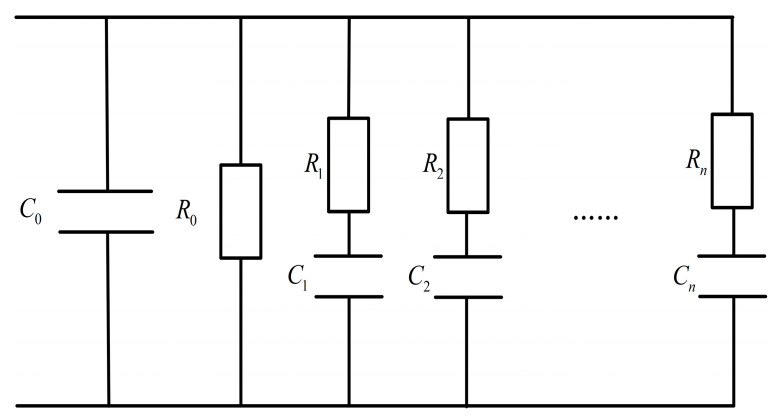
Extended Debye model of XLPE insulation.

XLPE is cross-linked by LDPE, crosslinking agent, and antioxidant. When an external electric field is applied to XLPE, different polarization types such as volume polarization, interface polarization, dipole orientation polarization, etc., [20] lead to different relaxation processes. It will return to its original state after the external electric field is withdrawn. To describe this relaxation phenomenon of dielectric, Debye proposed an equivalent circuit model with multiple R-C branches in series, as shown in Figure 2. *R*_0_ and *C*_0_ represent the insulation resistance and vacuum capacitance of the XLPE insulation system, respectively. *R_i_* and *C_i_* are, respectively, the capacitance and resistance of every branch. All resistance and capacitance can be measured directly or calculated by PDC. Time constants and current amplitude of each branch contain abundant information about insulation deterioration.

## 3. Experimental Design

### 3.1. Sample Preparation and Pre-treatment

**Figure 3:**
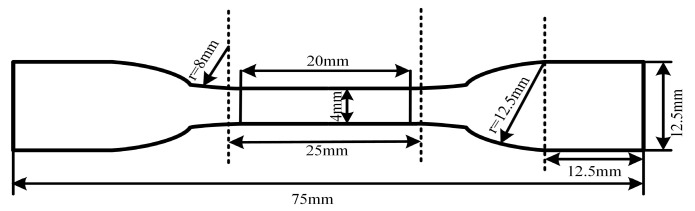
Dumbbell sample size diagram.

The condition of XLPE insulation layer of the cable has a significant impact on how well it performs. XLPE insulation film and dumbbell samples are prepared by plasticizing and vulcanizing 110 kV cross-linked polyethylene particles made in China according to industrial standards. The loss factor of XLPE is 3 × 10^−4^ at 25 °C, the elongation at break is 570.45%, and the dielectric constant is 2.21. The thickness of the disk is 1 mm and the diameter is 16 cm. Figure 3 is a size diagram of the dumbbell sample. The prepared sample is cleaned with anhydrous ethanol to remove the surface impurities of the sample. The treated samples are then dried in a vacuum drying oven for 48 h. In order to eliminate the impact of water and cross-linked by products, the vacuum drying oven is set at 90 °C and 50 Pa of pressure.

### 3.2. Accelerated Thermal Ageing of XLPE

The pre-treated disk samples and dumbbell samples are put in the ageing chamber for thermal ageing. According to IEC60216-1 standard, the ageing temperature is 150(±0.1) °C, and the duration of thermal ageing is 48 days. The ageing temperature is 150(±0.1) °C, and the duration of thermal ageing is 48 days. To stop the XLPE from adhering to the drying rack, a high-temperature anti-sticking cloth is positioned between them. Samples are taken at 6 days, 12 days, 24 days, and 48 days.

### 3.3. Scanning Electron Microscope(SEM) Test

The cross-section of the specimens is observed by SEM using a SU8020 scanning electron microscope (Hitachi High-Technologies Corp., Tokyo, Japan). The specimens are sprayed with gold. Ion sputtering instrument is used to spray gold on the observed side of the aged sample.

### 3.4. PDC and Mechanical Measurement

**Figure 4:**
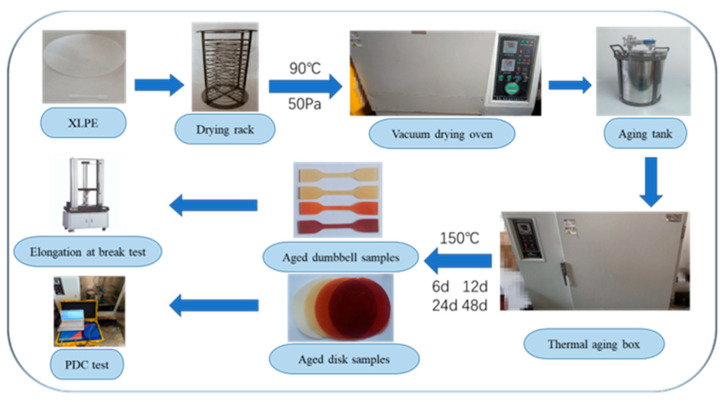
Experiment details of thermal aging and test.

The disk sample is used for PDC measurement. The three-electrode measuring device designed in this paper can eliminate leakage current well and improve the accuracy of data. The measuring equipment is DIRANA produced by Omicron, Austria. The excitation voltage applied is 200 V, where the polarization time and depolarization time are both 1500 s. The three-electrode measuring instrument is put in a metal closed constant temperature oven with a measurement temperature of 45 °C to eliminate the influence of outside noise. Due to the large fluctuation in the initial period of measurement, data with measuring time from 10 s to 1500 s are selected for analysis. The dumbbell samples are tested by UT-2080 tensile machine(U-CANUT-2080, Taiwan, China), and the stretching speed is 10 cm/min according to the IEC60811-501 standard. Three dumbbell samples are measured at each ageing time, and the average value of the three measurements is taken as the final result. The experimental details are shown in Figure 4.

## 4. Results and Analysis 

### 4.1. SEM Analysis

**Figure 5:**
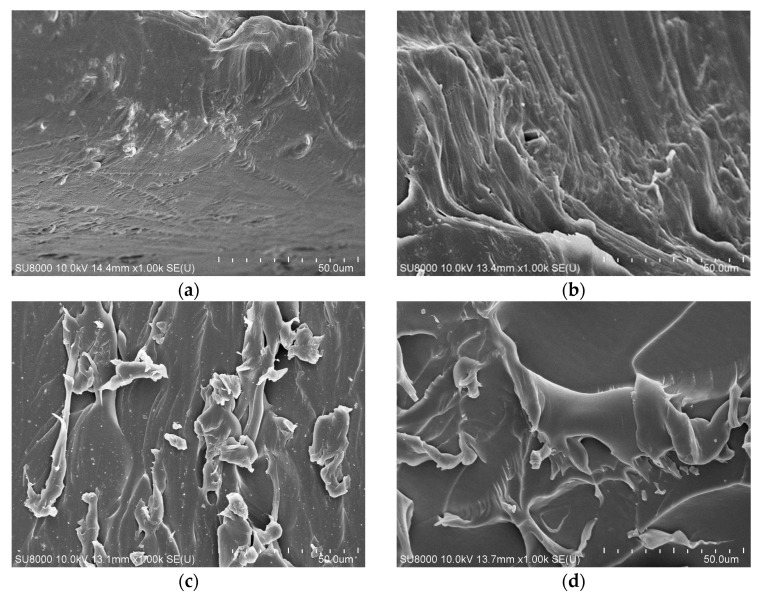
SEM results of XLPE with different ageing time. (**a**) 6 days. (**b**) 12 days. (**c**) 24 days. (**d**) 48 days.

Cross-linked polyethylene has a network structure. Every sample selects a microscopic morphology with defects and observes the microscopic morphology changes under different aging time, as shown in Figure 5.

Figure 5a is an SEM photo with aging time of 6 days. It shows smooth and dense structure, but there are also some small cracks. This can be observed from Figure 5b–d, with the thermal aging, the XLPE crystal structure deteriorated significantly, the cracks increased significantly, and the dense structure is destroyed. This change will affect the dielectric and mechanical properties of XLPE.

### 4.2. Polarization and Depolarization Current

**Figure 6:**
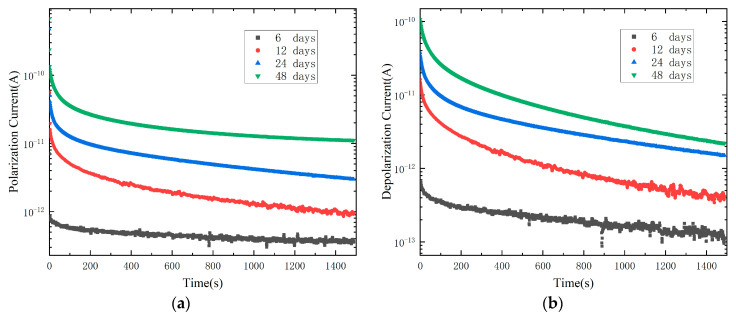
PDC measurement of XLPE samples with different ageing time. (**a**) Polarization current; (**b**) Depolarization current.

The polarization current and depolarization current with different ageing time, as shown in Figure 6. It can be found that with the increase in ageing time, polarization current and depolarization current both increase significantly. The curve moves upward as a whole because thermal ageing will cause thermal degradation of XLPE macromolecules. The macromolecular chains of XLPE are broken and degraded to produce more polar groups [21], and the number of dipoles will increase, which is manifested in the increase in polarization current and depolarization current on a macro level. It can be found from the polarization current curve that the more seriously ageing the sample is, the earlier it reaches the relatively stable current value. This may be because the conductance current in the polarization current is the main component, and the conductance current is almost parallel to the time axis in a straight line, which may cover up some relaxation information.

### 4.3. Trap Parameters of XLPE Samples

There are inevitably a large number of defects in XLPE, which form different trap levels in the polymer. The depth and number of trap levels of XLPE will change after thermal ageing, so the ageing state of XLPE insulation can be evaluated by studying the changes of the trap distribution of XLPE.

The relationship between trap depth *W_T_*(*t*) and time t in a dielectric insulation system can be expressed by the following formula [22]:

(4) $$W_{T}(t)=kT\ln(vt)$$

where *k* is the Boltzmann constant, *ν* is the attempt to escape frequency, and *T* is thermodynamic temperature.

According to the literature [22], the change of depolarization current over time can be given by the following:

(5) $$i_{dep}(t)=\frac{edkT}{2t}Q_{0}(W)N(W)$$

Here, *e* is the charge, *d* is the insulation thickness. *Q*_0_(*W*) is the initial density of the traps by electrons, and *N*(*W*) is the density of trap level distribution.

It can be found from (5) that the product of depolarization current and time can indirectly reflect the density of trap levels corresponding to the depolarization current. The distribution of electron trap above Fermi level can be determined by the profile of *i_dep_* × *t* vs. *log*_10_*t*.

**Figure 7:**
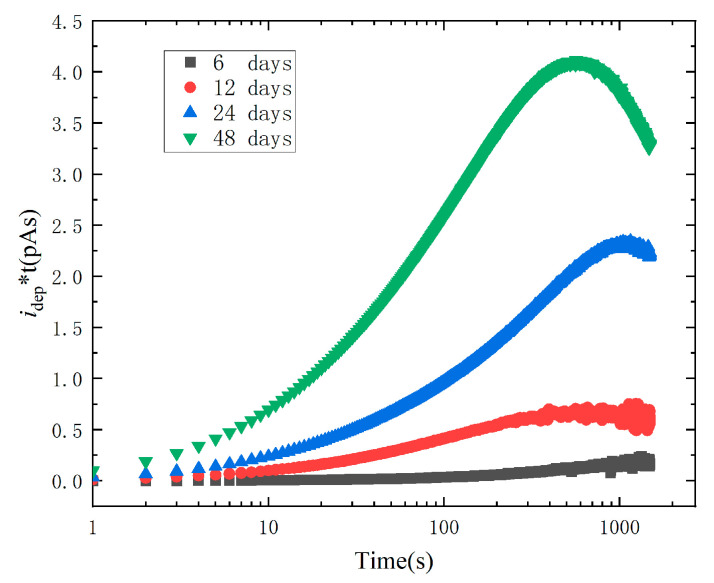
*i_dep_*(*t*) × *t* vs. *log*_10_*t* curves.

Figure 7 depicts the correlation between the trap level distribution and trap depth. All ageing samples have obvious peaks. The peak value increases with ageing time, indicating that more space charges are trapped in XLPE insulation during the polarization stage and more traps are produced in XLPE insulation. The peak time shifts to the left, which may be caused by the increase in the deep trap in XLPE after thermal ageing.

### 4.4. Conductivity

When the insulation polarization time of XLPE is long enough, the conductivity of XLPE can be determined by the following formula:

(6) $$\delta_{0}\approx \frac{\varepsilon_{0}}{C_{0}U_{0}}[i_{pol}(t)-i_{dep}(t)]$$

The sample geometric capacitance *C*_0_ is the capacitance value at 50 Hz [23], where (*i_pol_*(*t*) − *i_dep_*(*t*)) should be the steady-state conduction current value. In this paper, the average value of the difference between polarization and depolarization current of the last 10 s is taken. The calculated conductivity of ageing 6 days, 12 days, 24 days, 48 days is 1.25 × 10^−16^ S/m, 2.14 × 10^−16^ S/m, 1.21 × 10^−15^ S/m, 6.50 × 10^−15^ S/m, respectively.

For XLPE insulation, it can be found that the higher the conductivity, the more serious the thermal ageing state of XLPE insulation. There are two reasons make the increase of conductivity. On the one hand, the thermal oxidation reaction takes place inside XLPE, resulting in the generation of polar groups such as carbonyl groups [1]. Under the action of intrinsic dissociation, conductive ions can be produced. On the other hand, with the process of thermal ageing, the internal antioxidants of XLPE samples are almost consumed and the oxidative cracking process begins. The spherulites in XLPE begin to degenerate into lamellar crystals, which results in the crystalline zone transforming into the amorphous zone. The expansion of the XLPE lattice makes the structure looser [24], which will lead to the increase in amorphous region and ion mobility.

### 4.5. Elongation at Break Retention Rate (ER%)

**Figure 8:**
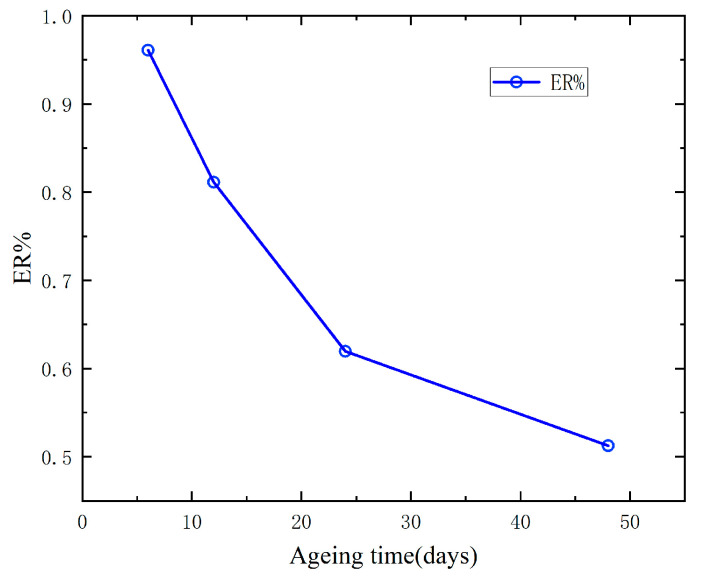
Elongation at break retention rate of dumbbell samples.

Under the influence of persistent high temperatures, XLPE insulation will experience oxidation, thermal cracking, and other chemical reactions, which will cause insulation materials crack and carbonization of the insulation and decrease in tensile strength. The tensile strength of XLPE insulation represents the limit of the material’s resistance to tensile failure, while the elongation at break represents the characteristics of the material network. Theseare the basic performance indexes of XLPE insulating materials. For the ageing degradation of XLPE insulation, both the tensile strength and elongation at break will decrease with the increase of thermal ageing time. Therefore, ER% is used as the evaluation standard of the thermal ageing state of XLPE insulation in this paper. The ER% can be obtained by the following formula:

(7) $$ER\%=\frac{k}{k_{0}}\times 100\%$$

where *k* is the elongation at break of XLPE samples with different ageing, and *k*_0_ is the elongation at break of XLPE dumbbell samples without ageing. The value is 570.45%. The ER% of XLPE samples with different ageing time at 150 °C can be obtained, as shown in Figure 8.

As shown in Figure 8, ER% decreases with continuous thermal ageing. The changeing trend of ER% in the early stage is steeper than that in the later stage. When the thermal aging time reaches 48 days, ER% is 50.12%, which is close to the end of XLPE insulation life [25]. Therefore, in this paper, ER% is to characterize the thermal aging state of XLPE. However, the measurement of ER% is a destructive experiment and is not suitable for field evaluation. The following chapter will dig out the characteristic parameters related to ER% from PDC.

### 4.6. Parameter Identification of Extended Debye Model

The parameters of the extended Debby model in Figure 2 can be obtained from the measured polarization and depolarization current. Each R-C branch in series in the figure can represent an independent dipole polarization process. For polymer insulation, the depolarization current, resistance, and capacitance values of the branch can be described by the following formula:

(8) $$i_{dep}(t)=\sum_{i=1}^{n}A_{i}e^{-\frac{t}{\tau_{i}}}$$

(9) $$R_{i}=\frac{U_{0}}{A_{i}}(1-e^{-\frac{t_{c}}{\tau_{i}}})$$

(10) $$C_{i}=\frac{\tau_{i}}{R_{i}}$$

where *n* represents the number of R-C series branches of the extended Debye model, *A_i_* is the relaxation coefficient of branch *i τ_i_* is the relaxation time constant of the *i* branch.

In order to identify model parameters, the matrix pencil algorithm (MP) is used to identify the parameters of the extended Debye model in this paper. This method constructs the Hankle matrix through the depolarizing current. The number of relaxation branches is uniquely determined by this method from the number of singular values of the matrix. Then, the current amplitude and relaxation time constant of each branch are calculated on this basis. The matrix pencil algorithm can well reflect the physical meaning of the extended Debye model, and can also effectively identify data containing noise [26].

The extended Debye model parameters of XLPE insulation samples with different ageing time are shown in Table 1, Table 2, Table 3 and Table 4. According to the result, the relaxation branch of the extended Debye model will increase as the ageing time grows, which indicates that new polar groups appear in the XLPE samples under continuous thermal ageing. After thermal ageing, the XLPE sample has lower resistance and capacitance values.

## 5. Quantitative Evaluation of Thermal Aging State from PDC

### 5.1. Time Domain Characteristic Parameter: Stable relaxation Charge Quantity (Q_s_)

Since the trap density and depth of XLPE increase after thermal ageing, more charge will be captured during polarization. The parameters identified in Table 1, Table 2, Table 3 and Table 4 can be calculated according to the following formula to obtain the relaxation charge of XLPE samples with different ageing time.

(11) $$Q_{r}(t)=\int_{0}^{t}\sum_{i=1}^{n}A_{i}e^{-\frac{t}{\tau_{i}}}dt$$

where *t* is the relaxation time, 0 ≤ *t* ≤ 1490 s.

**Figure 9:**
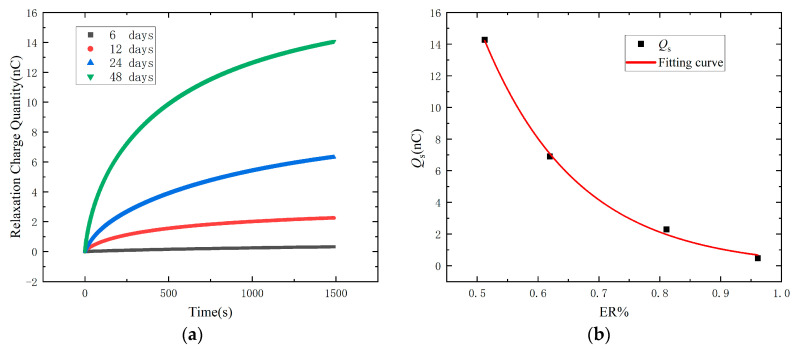
(**a**) Relaxation charge quantity with different ageing time; (**b**) Fitting curve between *Q*_s_ and the thermal ageing state.

Figure 9a shows the relaxation charge quantity of XLPE samples at different ageing times. It can be found that the relaxation charge quantity is very sensitive to the ageing state of XLPE insulation samples. As the thermal aging time grows, the amount of relaxation charge of the XLPE insulation sample also increases, and its time to reach a steady state is longer. The relationship between relaxation charge quantity and measurement time is shown in Figure 9b, with the regression coefficient reaching more than 0.993. For each XLPE insulation sample, the relaxation charge quantity is exponentially relevant to the measurement time. *Q_r_*_1_(*t*) represents the relaxation charge quantity of the XLPE insulation sample with an ageing time of 6 days, *Q_r_*_2_(*t*) represents the relaxation charge quantity of the XLPE insulation sample with an ageing time of 12 days, etc.

In Table 5, the constant *A* is the stable relaxation charge quantity. The stable relaxation charge quantity of XLPE insulation samples with different aging times can be obtained from the table. In order to quantitatively evaluate the aging state of XLPE insulation, the relationship between the stable relaxation charge quantity and the ER% is studied. From Figure 9b and Table 6, the stable relaxation charge quantity proposed in this paper can reflect the ER% of XLPE insulation and further forecast the thermal ageing state of XLPE insulation.

### 5.2. Frequency Domain Characteristic Parameter: Dissipation Factor at 0.1 Hz (tanδ_0.1Hz_)

In the frequency domain, the measuring time required in very low frequency is longer. However, the measurement time in very low frequency can be significantly reduced by calculating the dissipation factor from the time domain parameters. The dissipation factor of XLPE can be obtained from the resistance and capacitance value of the extended Debye model. The equation is shown as follows [27]:

(12) $$\tan\delta =\frac{C^{\prime \prime }}{C^{\prime }}=\frac{\frac{1}{\omega R_{0}}+\sum_{i=1}^{n}\frac{C_{i}}{1+{\left(R_{i}\omega_{i}C_{i}\right)}^{2}}}{C_{0}+\sum_{i=1}^{n}\frac{R_{i}{\left(\omega C_{i}\right)}^{2}}{1+{\left(R_{i}\omega_{i}C_{i}\right)}^{2}}}$$

**Figure 10:**
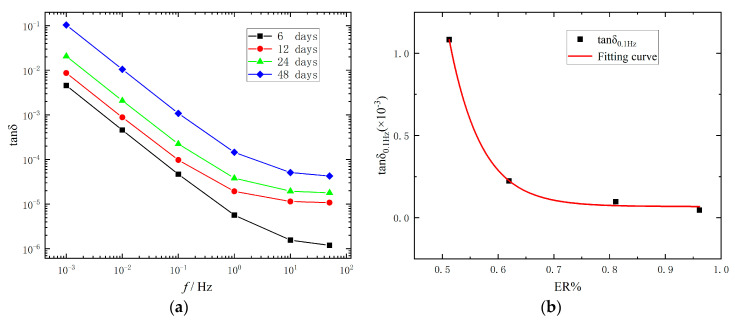
(**a**) Dissipation factor with different ageing time; (**b**) Fitting curve between tan*δ*_0.1Hz_ and the thermal ageing state.

The variation of dissipation factor with frequency obtained from the Formula (12) is shown in Figure 10a, and it can be seen that the dissipation factor of XLPE will increase with thermal aging, and the dissipation factor curve will decline slowly in high frequency. In Figure 10b and Table 6, there is a strong correlation between the dissipation factor at 0.1 Hz and ER%.

## 6. Conclusions

This paper confirms the validity of PDC as a non-destructive evaluation of the thermal aging state. The insulation condition of thermal ageing is determined by ER%. The characteristic parameters *Q*_s_ and tan*δ*_0.1Hz_ are extracted from PDC, and the quantitative relationship between the characteristic parameters and the ER% is established.

1. Based on the PDC measurement, both polarization current and depolarization current gradually increase with the thermal aging degree of the XLPE insulation, which has a good consistency in characterizing the deterioration of the XLPE insulation, and can make a preliminary assessment of the XLPE insulation state, along with ageing time and the conductivity increments.
2. The depolarization current data at the different ageing duration are applied to plot the *i_dep_*(*t*) × *t* vs. *log*_10_*t* curves which reflect the information about the changes of trap depth and density.
3. The number of relaxation branches of the extended Debye model will increase with ageing time, indicating that new relaxation types have emerged. The resistance and capacitance of XLPE will also decrease with thermal ageing.
4. ER% decreases with ageing time. It dwindles to 50.12% after 48 days ageing, which is close to the end of XLPE insulation life. The thermal ageing state can be determined by ER%.
5. Relaxation charge quantity and tan*δ* calculated by PDC are increasing with the increase of ageing degree. *Q_s_* and tan*δ*_0.1Hz_ proposed in this paper have a strong correlation with the ER%, which are expected to be a potential tool for quantitatively evaluating the ageing state.

## Figures and Tables

**Table 1:** Parameters identification of 6-day XLPE sample.

Branch	*A*i (A)	*τ*i (s)	*R*i (Ω)	*C*i (F)
1	2.50 × 10^−13^	45.80	8.01 × 10^14^	5.72 × 10^−14^
2	3.33 × 10^−13^	1418.13	3.92 × 10^14^	3.62 × 10^−12^

**Table 2:** Parameters identification of 12-day XLPE sample.

Branch	*A*i (A)	*τ*i (s)	*R*i (Ω)	*C*i (F)
1	6.59 × 10^−12^	7.99	3.03 × 10^13^	2.63 × 10^−13^
2	4.37 × 10^−12^	53.35	4.58 × 10^13^	1.17 × 10^−12^
3	3.48 × 10^−12^	252.10	5.73 × 10^13^	4.40 × 10^−12^
4	1.22 × 10^−12^	1328.03	1.11 × 10^14^	1.20 × 10^−11^

**Table 3:** Parameters identification of 24-day XLPE sample.

Branch	*A*i (A)	*τ*i (s)	*R*i (Ω)	*C*i (F)
1	7.12 × 10^−12^	3.87	2.81 × 10^13^	1.38 × 10^−13^
2	1.04 × 10^−11^	15.27	1.92 × 10^13^	7.94 × 10^−13^
3	9.37 × 10^−12^	55.99	2.13 × 10^13^	2.62 × 10^−12^
4	5.06 × 10^−12^	216.16	3.95 × 10^13^	5.47 × 10^−12^
5	5.41 × 10^−12^	1147.95	2.69 × 10^13^	4.26 × 10^−11^

**Table 4:** Parameters identification of 48-day XLPE sample.

Branch	*A*i (A)	*τ*i (s)	*R*i (Ω)	*C*i (F)
1	1.83 × 10^−11^	4.03	1.09 × 10^13^	3.69 × 10^−13^
2	3.23 × 10^−11^	15.45	6.19 × 10^12^	2.49 × 10^−12^
3	2.61 × 10^−11^	52.66	7.66 × 10^12^	6.87 × 10^−12^
4	1.95 × 10^−11^	211.73	1.02 × 10^13^	2.06 × 10^−11^
5	1.09 × 10^−11^	918.19	1.48 × 10^13^	6.22 × 10^−11^

**Table 5:** Relation between the relaxation charge quantity and measuring time.

Ageing Time (Days)	Fitting Formula $Q_{r}(t)=A+B\times \exp(-t/C)$	*R* ^2^
6	$Q_{r1}(t)=4.71009\times 10^{-10}-4.62258\times 10^{-10}\times \exp\left(-t/1352.7301\right)$	0.999
12	$Q_{r2}(t)=2.29494\times 10^{-9}-2.09095\times 10^{-9}\times \exp(-t/489.54102)$	0.993
24	$Q_{r3}(t)=6.90548\times 10^{-9}-6.27955\times 10^{-9}\times \exp\left(-t/681.3204\right)$	0.996
48	$Q_{r4}(t)=1.42751\times 10^{-8}-1.28663\times 10^{-8}\times \exp\left(-t/469.40095\right)$	0.993

**Table 6:** Fitting Equation between characteristic parameter and ER%.

Ageing Time (Days)	Fitting Formula	*R* ^2^
*Q*_s_	$Q_{s}=3.9074\times 10^{-7}\times \exp(-\mathrm{ER}\%/0.1549)-1.1295\times 10^{-10}$	0.995
tan*δ*_0.1Hz_	$\tan\delta_{0.1Hz}=7.49429\times \exp(-\mathrm{ER}\%/0.05752)+6.80947\times 10^{-5}$	0.998

## Data Availability

The study did not report any data.
